# Exploring the association of respiratory infection aetiology with mental well-being scores: a longitudinal study

**DOI:** 10.3389/fpsyg.2026.1833497

**Published:** 2026-08-10

**Authors:** Abigail Murfitt, Alike W. van der Velden, Femke Böhmer, Christopher C. Butler, Slawomir Chlabicz, Annelies Colliers, Ana Garcia-Sangenis, Lile Malania, Jozsef Pauer, Angela Tomacinschii, Akke Vellinga

**Affiliations:** 1 University College Dublin School of Public Health, Physiotherapy and Sports Science, Dublin, Ireland; 2 Julius Centrum voor Gezondheidswetenschappen en Eerstelijns Geneeskunde, Utrecht, Netherlands; 3 Universitat Rostock, Rostock, Germany; 4 University of Oxford Nuffield Department of Primary Care Health Sciences, Oxford, United Kingdom; 5 Department of Family Medicine, Medical University of Bialystok, Bialystok, Poland; 6 Department of Family Medicine and Population Health, University of Antwerp, Antwerp, Belgium; 7 Institut d'Investigacio en Atencio Primaria de Salut Jordi Gol, Barcelona, Spain; 8 National Center for Disease Control and Public Health, Tbilisi, Georgia; 9 DRC Drug Research Centre, Veszprém, Hungary; 10 Universitatea de Stat de Medicina si Farmacie Nicolae Testemitanu, Chișinău, Moldova

**Keywords:** infection types, mental well-being, post infection well-being, SARS-CoV-2, well-being

## Abstract

**Background:**

Acute respiratory infections can disrupt both physical and psychological well-being. This study aimed to examine whether mental well-being differed according to respiratory infection aetiology during the first year of the SARS-CoV-2 pandemic and to identify factors associated with mental well-being over time.

**Methods:**

This prospective observational cohort study included 819 adults from nine European countries recruited during 2020 and 2021 as part of the SOS-COVID study. Mental well-being was assessed using the WHO-5 Well-Being Index at day 7 and day 28 following enrolment. Respiratory infection aetiology was classified as SARS-CoV-2, other viral infection, or no virus detected, and additionally grouped as SARS-CoV-2 versus no SARS-CoV-2 detected. Linear mixed-effects models with patients nested within countries examined associations between infection type and well-being scores, adjusting for age, recovery duration and time period.

**Results:**

Median WHO-5 well-being scores increased from 13 at day 7 to 18 at day 28. Participants with SARS-CoV-2 infection reported lower median well-being scores (12 and 17 at day 7 and day 28, respectively) compared with other viral infections (15 and 19), non-SARS-CoV-2 infections (13 and 18), and no virus detected (13 and 17). In adjusted analyses, lower day 28 well-being was associated with longer recovery duration (coef = −0.1, 95% CI: −0.2 to −0.1), SARS-CoV-2 infection (coef = −1.1, 95% CI: −1.8 to −0.3), recruitment during July to September and October to December 2020, and increasing age. Approximately 32% of variance in well-being occurred at the country level.

**Conclusion:**

SARS-CoV-2 infection was associated with lower mental well-being scores, particularly 1 month following enrolment, compared with other respiratory infection groups. Recovery duration and recruitment period also influenced well-being outcomes, suggesting that both infection-specific and contextual pandemic-related factors contributed to reduced mental well-being.

## Introduction

Acute respiratory infections are amongst the most common infectious diseases worldwide and represent a substantial cause of morbidity and healthcare utilisation and frequently disrupt daily life and mental well-being (Smith, 2013). Since its emergence in late 2019, SARS-CoV-2 has caused over 770 million confirmed cases and more than 7 million deaths worldwide, highlighting a substantial global public health impact (World Health Organisation, 2020). Regardless of the type of respiratory infection, various physical and psychological problems can arise, ultimately affecting both physical and mental well-being in affected individuals (Angner et al., 2009).

The impact of acute respiratory infections on well-being has also been demonstrated in community influenza cohorts. An English study found influenza and influenza-like illness were associated with reductions in health-related quality of life, impairment in usual activities, and loss of quality-adjusted life days during acute illness (Fragaszy et al., 2018). Similarly, studies of individuals recovering from SARS-CoV-2 infection have reported reduced quality of life and poorer mental health outcomes beyond the acute phase of infection (Xie et al., 2022). Although many of these effects are linked to the acute symptomatic phase, respiratory infections may also have broader implications for mental well-being. Prior studies comparing different categories of respiratory illness presentation have focused on acute quality of life (Bilcke et al., 2014), with limited evidence regarding whether outcomes differ according to infection type.

The interplay between respiratory infections and mental well-being became particularly visible during the SARS-CoV-2 pandemic, during which anxiety specifically related to SARS-CoV-2 (“coronaphobia”) was associated with higher levels of generalised anxiety, death anxiety and depression (Lee et al., 2020). However, whether mental well-being differs according to respiratory infection aetiology remains unclear. This study aimed to examine whether mental well-being differed according to respiratory infection aetiology in a European cohort of patients who presented to their GP with symptoms of acute respiratory infection during the first year of the SARS-CoV-2 pandemic, with particular focus on SARS-CoV-2, other viral infections and cases in which no virus was detected.

## Methods

Data was collected as part of the SOS-COVID study, a prospective observational cohort study across nine European countries during the first year of the SARS-CoV-2 pandemic (2020–2021) (van der Velden et al., 2023). General practises in Belgium, Germany, Spain, Georgia, Hungary, Ireland, Moldova, the Netherlands and Poland participated in this study. Ethical approval was obtained separately in each participating country.

Patients presenting to their GP with symptoms of a respiratory tract infection were invited to participate. Eligibility criteria for the original study were aged 1 year or older, presence of cough, sore throat and/or rhinitis, with symptoms less than 14 days, and unknown disease aetiology. Although the original SOS-COVID study included participants aged under 18, the present secondary analysis was restricted to participants 18 and above, to ensure that mental well-being was assessed using a validated self-reported measure appropriate for the study population.

Patients were enrolled during face-to-face, telephone or virtual consultations. Informed consent was obtained from all patients prior to study enrolment. GPs completed a case report form which detailed patient characteristics, comorbidities, sign and symptoms and risk factors. A combined oropharyngeal and nasal swab was taken during the consultation, or where this was not feasible, collected at home by practise personnel or self-collected following study instructions. Swabs were analysed in the central laboratory in Antwerp, Belgium. Formal assessment of sample quality or comparability between collection methods was not performed. Trained researchers conducted follow-up interviews by phone on day 7 and day 28. Patients answered questions about their overall recovery and recovery from specific symptoms and mental well-being was measured using the WHO (five) Well-Being Index. Five well-being questions asked participants on the frequency of cheerfulness, calmness, activeness, freshness and interest in the previous 2 weeks, ranging from ‘at no time’ to ‘all of the time’ (World Health Organisation, Regional Office for Europe, 1998). The total well-being score ranged between 0 (worst possible mental well-being) and 25 (best possible mental well-being). Loss to follow-up occurred due to non-response to telephone assessment, and analyses at each time point included only participants with available outcome data.

Swabs were analysed for a panel of viral and bacterial pathogens (Bilcke et al., 2014). Bacterial infections were not considered in this analysis, due to the high presence of positive, potential carriership, bacterial swabs. Respiratory infection aetiology was classified using two grouping schemes for separate analyses: (1) a three-group classification comprising SARS-CoV-2 infection, other viral infection (mainly rhinovirus) and no virus detected; and (2) a binary classification of SARS-CoV-2 versus no SARS-CoV-2 detected. Participants with SARS-CoV-2 were classified in the SARS-CoV-2 group regardless of co-infection, whilst participants with one or more non-SARS-CoV-2 viral pathogens were classified as other viral infections.

Statistical analyses were conducted in IBM SPSS 29 and RStudio. Linear mixed-effects models with patients nested within countries were calculated for day 7 and day 28 mental well-being outcomes. Models included age, observed recovery duration, time period and type of infection as fixed effects, with WHO-5 well-being scores as the outcome variable, with country specified as a random intercept to account for clustering at the country level. Recovery duration referred to overall recovery from the respiratory tract infection and was calculated by the interviewer based on patient-reported day of full recovery and symptom onset date collected during follow-up telephone interviews. As participants were eligible only if symptoms had been present for less than 14 days at enrolment, the maximum observable recovery duration was 42 days. Recovery duration was modelled as a continuous variable representing days until full recovery. Participants without recorded recovery duration were excluded from the main analyses due to ambiguity regarding whether missing values reflected non-response or lack of recovery. Sensitivity analyses applying an alternative coding in which missing values represented non-recovery or recovery beyond follow-up are presented in Supplementary Table S1. A time period variable was created by dividing recruitment into consecutive three-month periods (April to June 2020, July to September 2020, October to December 2020, and January to March 2021). These periods were selected to account for temporal variation in recruitment occurring during different phases of the SARS-CoV-2 pandemic across participating countries and to account for potential changes in healthcare context, public health restrictions and circulating respiratory infections over time. Missing data were handled using complete-case analysis, whereby participants with missing outcome or predictor data relevant to a given model were excluded from that specific analysis.

Assumptions of normality and homogeneity of variance were assessed before proceeding with analyses. Normality was evaluated using the Shapiro–Wilk test together with visual inspection of histograms and Q-Q plots. Although the Shapiro–Wilk test indicated statistically significant deviations from normality across groups on both days, visual inspection suggested only minor deviations. Given the large sample size, parametric tests were considered appropriate, as they are robust to modest violations of normality. Homogeneity of variances was assessed using Levene’s test and was satisfied for all comparisons on both days. For continuous predictors included in the multilevel models, linearity was assessed through visual inspection and comparison of linear and quadratic model specifications. No substantial deviations from linearity were observed for age or recovery duration. Statistical significance was defined as a two-sided *p*-value <0.05.

## Results

A total of 819 participants from 9 countries were included (Table 1). The median age was 40 years, from 36 in Germany to 49 in the Netherlands. Up to 70% of the patients in Hungary were female and 46% in Ireland, with overall 57% female and 43% male.

**Table 1:** Overview of demographics and mental well-being scores of enrolled patient by country.

Country	*n*	Age	Gender		Full recovery^1^	Not recovered^2^	Well-being day 7^3^	Well-being day 28^3^
		Median (IQR)	Male *n* (%)	Female *n* (%)	Median (IQR)	*n* (%)	Median (IQR)	Median (IQR)
Belgium	93	37 (31–49)	49 (53%)	44 (47%)	5 (3.2–10)	24 (26%)	14 (10–18)	18 (15–20)
Germany	95	36 (30–49.5)	49 (52%)	46 (48%)	5 (3–7)	12 (13%)	13 (10–18)	17 (11.5–19)
Spain	81	39 (28–54)	41 (51%)	40 (49%)	6 (3–12)	12 (15%)	10.5 (8.2–13)	15 (12–19)
Georgia	144	38 (29–50.2)	47 (33%)	97 (67%)	9 (7–10.5)	9 (6%)	12 (9–15)	15 (15–17)
Hungary	100	44 (30–53.2)	30 (30%)	70 (70%)	9 (6–13)	4 (4%)	13 (11–15)	21 (19–23)
Ireland	44	38 (24.8–48.2)	24 (55%)	20 (45%)	4 (3–7)	14 (32%)	15.5 (11–17)	18 (14.2–20)
Moldova	96	38 (30–48)	42 (44%)	54 (56%)	10 (6–14)	3 (3%)	12 (10–16)	19 (15–20)
Netherlands	91	49 (44–57)	45 (50%)	46 (50%)	10 (6–20)	56 (62%)	10 (6–15)	18 (14–20)
Poland	75	37 (32.5–44)	28 (37%)	47 (63%)	6 (3–7)	9 (12%)	20 (17–22.8)	24 (20–25)
Overall	819	40 (30–51)	355 (43%)	464 (57%)	7 (5–11)	143 (18%)	13 (10–17)	18 (15–20)

^1^Days until fully recovered from illness. ^2^Not recovered by interview on day 28. ^3^5 questions scored 0–5, higher scores indicate increased mental well-being.

The overall median time to recovery was 7 days but varied widely across countries, with Ireland having the shortest median time (4 days) and the Netherlands and Moldova the longest (10 days). By day 28, 18% of patients had not indicated being fully recovered from their respiratory tract infection, with again a wide variety between countries, from 62% in the Netherlands to 3% in Moldova.

The WHO-5 Well-Being Index overall median score was 13 on day 7 and 18 on day 28, with good internal consistency on both days (Cronbach’s alpha = 0.880 on day 7, and 0.911 on day 28). The Netherlands had the lowest median well-being score (10) on day 7, whilst Georgia’s and Spain’s median score (15) was the lowest on day 28. Poland reported the highest well-being scores on both days, with median scores of 20 and 24 on day 7 and day 28, respectively.

Patients with a SARS-CoV-2 infection had generally lower median scores (12 and 17 on day 7 and 28) compared to other viral infections (15 and 19 on day 7 and 28), or to non-SARS-CoV-2 infections (13 and 18 on day 7 and 28) and no virus detected (13 and 17 on day 7 and 28).

The multilevel model (Table 2) showed that a lower well-being score 1 month after enrolment was associated with longer recovery time (coef = −0.1, 95% CI: −0.2 to −0.1), recruitment later in the pandemic (July to September and October to December 2020 (coef = −1.6, 95% CI: −2.7 to −0.4) and (coef = −1.4, 95% CI: −2.4 to −0.3) respectively), a confirmed SARS-CoV-2 infection (coef = −1.1, 95% CI: −1.8 to −0.3) and increased age (coef = −0.0, 95% CI: −0.1 to −0.0). A three group infection model was conducted which compared no virus detected infections against COVID-19 infections and other viral infections. Whilst SARS-CoV-2 infection was significantly associated with lower well-being scores in this analysis, (coef = −0.9, 95% CI: −1.7 to −0.0), this was not detected for other viral infections (coef = −0.6, 95% CI: −0.3 to 1.5).

**Table 2:** Final multilevel model results for SARS-CoV-2 on day 28.

Measure	coef	L-CI	U-CI
Intercept	21.4	19.2	23.6
Age	−0.0	−0.01	−0.0
Recovery time	−0.1	−0.2	−0.1
April to June 2020	REF		
July to September 2020	−1.6	−2.7	−0.4
October to December 2020	−1.4	−2.4	−0.3
January to March 2021	−1.0	−2.5	0.5
SARS-CoV-2 positive	−1.1	−1.8	−0.3

Random effects	
ICC	32%
Country-level variance	6.9
Individual-level variance	14.9

**Figure 1:**
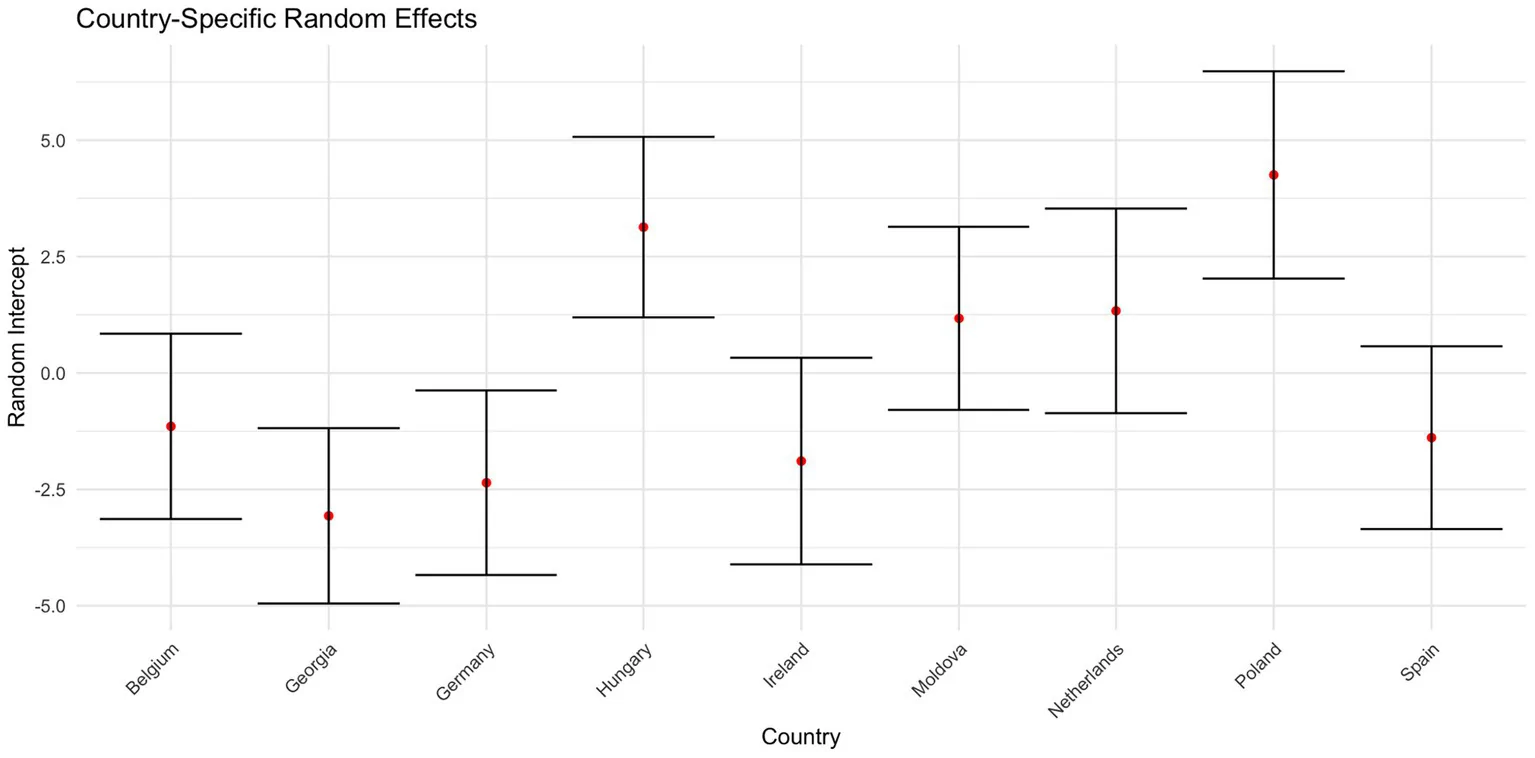
Country-specific random effects coefficients.

Figure 1 presents country-specific random effects (red dot), with the corresponding 95% confidence interval to indicate how much of the variance is due to country differences. The confidence intervals for Poland, Hungary (higher well-being scores), Georgia and Germany (lower well-being scores) do not cross zero, suggesting a statistical significant difference. Approximately 32% of the variance in well-being could be attributed to the country (Tables 1, 3; Figure 1).

**Table 3:** Well-being scores by infection type and time point.

Infection Type	*n*	Well-being day 7	*p* value*	*n*	Well-being day 28	*p* value*
		Median (IQR)			Median (IQR)	
SARS-CoV-2	189	12 (10–15)	<0.001	188	17 (14–20)	<0.01
Other viral	138	15 (12–17)		132	19 (15–22)	
No virus detected	449	13 (10–17)		443	17 (15–20)	
SARS-CoV-2	189	12 (10–15)	<0.05	188	17 (14–20)	0.33
No SARS-CoV-2 detected	587	13 (10–17)		575	18 (15–20)	

*p values represent overall group comparisons of WHO-5 well-being scores between infection groups at each time point.

## Discussion

This study showed a significant association between SARS-CoV-2 infection and lower mental well-being scores a week after consulting the GP and particularly after 1 month compared to those without SARS-CoV-2 infection, or other viral infections. In addition, well-being decreased with the ongoing pandemic and longer duration of symptoms.

SARS-CoV-2 infection was a significant predictor of lower well-being scores compared to Non-SARS-CoV-2 infection, even after controlling for age, recovery time, time of recruitment and country level differences. This suggests that SARS-CoV-2 carries a unique emotional burden and the prolonged effect on mental well-being could be explained in some cases by post-COVID syndrome (long-COVID) (Hedin et al., 2023). Consistent with these findings, Taquet et al. reported increased risks of mood and anxiety disorders following SARS-CoV-2 compared with other respiratory tract infections. Importantly, these effects were observed not only amongst individuals with severe disease, but also in non-hospitalised patients, suggesting that poorer outcomes related to well-being following SARS-CoV-2 infection are not restricted to severe illness (Taquet et al., 2021). Similar findings have also been reported in a large cohort study by Xie et al., which observed elevated risks of several mental health outcomes following SARS-CoV-2 infection, including amongst individuals who were not hospitalised (Xie et al., 2022).

The decline of mental well-being scores with the ongoing pandemic may be a result of the cumulative emotional toll of the pandemic, given participants were living under some form of restrictions for at least 3 months at the time of inclusion (Lyons et al., 2020). This, combined with the heightened fear during the SARS-CoV-2 pandemic, which added uncertainty of long-term outcomes and distress in response to the virus outbreak, led to higher levels of health anxiety (Bilcke et al., 2014; Sauer et al., 2020). Additional factors such as constant reminders of the impact of SARS-CoV-2 on personal health and long-covid becoming an issue across social media and other mediums may have reinforced these negative responses.

Other factors, pertaining to lockdown measures and number of SARS-CoV-2 cases, likely explain the variance between countries’ well-being scores. One cross country comparison reports Germany implemented an early lockdown with relatively strict restrictions which resulted in fewer deaths compared to other European countries, whilst Spain was slow to implement strict measures which resulted in one of the highest death tolls in Europe (Cascini et al., 2022). Both countries reported lower well-being scores. Whilst Poland implemented strict measures, they also reported a lower death toll compared to other western countries (Balmford et al., 2020), which may partly explain higher well-being scores on both day 7 and 28. Georgia did have strict lockdown measures (COVID-19 Pandemic—Georgia, 2023) and showed consistent low well-being scores which may suggest that differences between countries are not necessarily due to the pandemic and lockdown measures, but other factors not considered in this analysis. In the World Happiness Report 2024 (Helliwell et al., 2024), which ranks countries using individuals’ scores of dystopia, perceptions of corruption, generosity, freedom to make life choices, healthy life expectancy, social support and GDP per capita, Georgia scored 91 out of 143 countries listed and is the lowest ranked country included in this study. Country comparisons of well-being scores can be seen in Supplementary Figures S1 and S2.

Strengths of this study lie in the a considerable sample size, with patients from multiple European countries. Follow-up on day 7 and day 28 added to the short-term and long-term mental well-being impacts of acute respiratory infections, particularly SARS-CoV-2.

Bacterial infections were not considered in this study, which may have skewed results. Amongst positive bacterial swabs, a distinction could not be made between those who were colonised with bacteria, and those with bacteria involved in the acute infection. As a result, the no-virus-detected group consisted of individuals who either had no infection detected, individuals who were colonised with bacteria but not actively infected, and potentially, individuals who had active bacterial infections. In addition, respiratory samples were collected through different approaches, including clinician-collected and self-collected swabs, whilst formal assessment of sample quality or comparability between collection methods was not available. As a result, difference in sampling approach may have influenced pathogen detection and contributed to some misclassification of infection aetiology, particularly within the no-virus-detected group. This heterogeneity may have introduced variability and influenced mental well-being scores in this group.

Finally, whilst the WHO (five) Well-Being Index (World Health Organisation, Regional Office for Europe, 1998) demonstrated strong internal consistency in this study, mental well-being is influenced by multiple individual and contextual factors that may not be fully captured by this instrument. In addition, the WHO-5 assesses mental well-being over the preceding 2 weeks, therefore at day 7 follow-up, the recall period may have included both pre infection and illness-related experiences for some participants, particularly those with shorter symptom duration at enrolment. Furthermore, pre-infection baseline well-being was not available. Some missing follow-up data were present at day 7 and day 28, which may have introduced bias if participants with incomplete follow-up differed from those included in the analyses. In addition, the multivariable analyses were based on complete cases. Although sensitivity analyses were performed for missing recovery data, the mechanism underlying missing predictor data could not be formally assessed. Consequently, if missingness was not completely at random, some bias may have been introduced into the estimated associations.

Consequently, findings should be interpreted with caution given the inherent limitations of self-reported measures and the potential influence of unmeasured factors. Although country-level clustering was accounted for, clustering at the individual practise level could not be examined and may have contributed to residual variability in mental well-being outcomes.

Finally, interpretation of recovery duration should be considered with caution, as recovery timing was derived from participant report and retrospectively calculated by interviewers based on symptom onset and reported day of full recovery. Missing recovery values in the original dataset could not be distinguished between non-response and non-recovery. Main analyses therefore included participants with observed recovery duration only. Sensitivity analyses applying an alternative coding in which missing recovery values represented non-recovery or recovery beyond follow-up produced substantively similar findings for SARS-CoV-2 status and recovery duration, although associations with recruitment period were attenuated (Supplementary Table S1).

The findings suggest that SARS-CoV-2 patients report lower well-being scores during and at the end of their acute infection compared to those with other respiratory infections and that well-being declined further as the pandemic progressed.

## Data Availability

The original contributions presented in the study are included in the article/supplementary material, further inquiries can be directed to the corresponding author.
