# Supplementary material for: Exploring the association of respiratory infection aetiology with mental well-being scores: a longitudinal study
Source: Front Psychol. 2026 Aug 10;17:1833497. doi: 10.3389/fpsyg.2026.1833497 (PMC13500573; doi:10.3389/fpsyg.2026.1833497)
Supplement: Supplementary file 1 [file Supplementary_file_1.docx]

Supplementary figure S1

|  |  |  |
| --- | --- | --- |
|  |  |  |
|  |  |  |

Supplementary figure S2

|  |  |  |
| --- | --- | --- |
|  |  |  |
|  |  |  |

Supplementary Table S1

| Measure | coef | L-CI | U-CI |
| --- | --- | --- | --- |
| Intercept | 19.7 | 17.8 | 21.5 |
| Age | 0.0 | -0.1 | 0.0 |
| Recovery time | -0.1 | -0.1 | -0.1 |
| April to June 202 | -0.6 | -1.8 | 0.6 |
| October to December 2020 | -0.2 | -1.2 | 0.8 |
| January To March 2021 | -0.3 | -1.8 | 1.2 |
| SARS-CoV-2 positive | -1.2 | -1.9 | -0.4 |
|  |  |  |  |
| Random effects | | | |
| ICC | | 25.3 | |
| Country-level variance | | 5.8 | |
| Individual Variance | | 17.2 | |
